# Mandibular symphysis onlay bone graft with i-PRF versus xenograft for maxillary anterior alveolar ridge augmentation: A comparative clinical study

**DOI:** 10.1007/s44445-025-00027-w

**Published:** 2025-06-14

**Authors:** Nermine Ramadan Mahmoud, Amany Ahmed AlAraby, Wessam Ibrahim Shehab Eldin, Yasser Fekry Habaka

**Affiliations:** 1 Oral Surgery Department, Faculty of Dentistry, October 6 University, Giza, Egypt; 2 Oral Medicine, Periodontology & Oral Radiology Department, Faculty of Dentistry, October 6 University, Giza, Egypt; 3 Oral and Maxillofacial Surgery Department, Faculty of Dentistry, October 6 University, Giza, Egypt

**Keywords:** Esthetic zone, Ridge augmentation, Onlay graft, Chin graft, I-PRF, Ridge widening

## Abstract

There are numerous factors that can impact both the correction of jaw deficiencies and the success of a particular grafting material, including the histology and density of both the maxillary and mandibular bones and the grafting material itself. This study compares the clinical outcomes of grafted augmentations of the horizontal alveolar ridge of the anterior maxilla using mandibular symphysis onlay bone that was admixed with either injectable platelet-rich fibrin (i-PRF) or xenografts. Twelve adult patients with horizontal maxillary alveolar ridge deficiency were randomly divided into two groups of six patients each. Group I received mandibular symphysis onlay bone grafts mixed with i-PRF, while Group II received mandibular symphysis onlay bone grafts admixed with xenografts (InterOss anorganic cancellous granules). CBCT scans were used to measure alveolar ridge width and bone density both preoperatively and at 6-month follow-up. Both groups showed improvements in alveolar ridge width and bone density. The increase in measured bone width and density after 6 months in Group II was significantly greater than that in Group I (*p* = 0.040). Horizontal alveolar ridge augmentation using an onlay chin graft in combination with xenografts was successful and offered adequate bone quantity and quality.

## Introduction

Alveolar bone resorption can cause diminished tooth support and heightened mobility with potential exfoliation, while limiting prosthetic options. Such pathologies can compromise facial architecture, impairing both function and appearance. Several grafting methodologies have been developed to treat these problems, including autogenous bone grafts, allografts, xenografts, synthetic substitutes, and platelet-rich fibrin (PRF) augmentation (Horowitz et al. 2012). Autogenous bone grafts are the most common option due to their superior osteogenic, osteoinductive, and osteoconductive qualities (Pascawinata and Bakar 2022), and methods to accelerate healing currently include angiogenesis stimulation, enhanced cellular proliferation and differentiation, extracellular matrix production augmentation, inflammatory response modulation, and osteogenesis promotion (Bassir et al. 2018; Zhou et al. 2017; Titsinides et al. 2019).

Autogenous grafting commonly results in optimal osteogenesis, osteoinduction, and osteoconduction due the compatibility of osteoblasts, osteoprogenitor cells, and growth factors (Urban et al. 2013). However, this technique requires careful consideration of morbidities at the donor site, vascularity at the recipient site, and the stability of the graft, and it entails meticulous soft tissue management. Furthermore, complications can include persistent pain, protracted operative time, infection risk, and peripheral neuropathy (Hämmerle et al. 2008; Ohayon 2011).

Ideal grafting materials exhibit osteoinduction and osteoconduction, while offering mechanical stability and gradual resorption that is synchronized with osteogenesis (Pascawinata and Bakar 2022; Jo et al. 2018). Osteogenesis is derived from viable osteosynthetic bone cells; osteoconduction provides scaffolding for vascular ingrowth and cellular migration; and osteoinduction stimulates osteoprogenitor differentiation via growth factors, particularly bone morphogenetic proteins.

PRF, introduced by Choukroun in 2001 (Shah et al. 2017), has evolved into multiple variants:

- L-PRF contains leukocytes, platelets, and fibrin (2700–3000 rpm, 12 min).
- A-PRF requires reduced centrifugation speeds, yielding elevated growth factor concentrations.
- i-PRF is a liquid formulation prepared at low speeds.
- T-PRF uses titanium tubes for clot formation.
- H-PRF uses horizontal centrifugation.
- C-PRF undergoes compression to yield denser membranes (Shah et al. 2017; Kapse et al. 2019).

Although C-PRF provides a denser membrane with potentially longer-lasting effects, the additional compression step may affect the viability of some cellular components (Zhang et al. 2020; Karde et al. 2017).

Choosing between PRF subtypes should be based on the procedure, patient factors, and desired outcomes. The advantages of i-PRF include its injectable nature and rich composition of growth factors. Its preparation involves collecting venous blood in 10 ml plain glass tubes without anticoagulants, followed by the standard immediate centrifugation at 700 rpm (60 g) for 3 min, which segregates the blood components without fibrin polymerization, following Titsinides et al. (Titsinides et al. 2019). The resultant liquid i-PRF occupies the upper fraction of the tube and requires extraction by syringe before clotting begins and immediate use to maximize growth factor efficacy (Wang and Yeung 2017). Thus, using i-PRF with autogenous bone grafting offers no significant improvement in osteogenic parameters, healing time, or postoperative morbidity compared to autogenous grafting alone. This study compares the effectiveness, healing time, and postoperative outcomes of autogenous bone grafting with i-PRF to autogenous bone grafting with xenografts.

## Materials and methods

The present study was conducted on twelve adult patients selected from those attending the outpatient clinic, Department of Maxillofacial Surgery, Dentistry College, October 6 University with age range between 24—40 years. Following the CONSORT criteria(Schulz et al. 2010; Cuschieri 2019) and Helsinki declaration principles, inclusion parameters comprised horizontal alveolar ridge deficiency in the anterior maxilla (< 4 mm labio-palatal dimension), sufficient vertical ridge height relative to adjacent alveolar bone, exemplary oral hygiene, and presence of healthy or appropriately restored adjacent teeth framing gaps spanning 1–4 missing teeth. Exclusion parameters encompassed systemic pathologies, inadequate oral hygiene, contraindications to implant surgery, uncontrolled diabetes, recent irradiation, chemotherapy, immunosuppressive therapy within five years, active periodontitis, and psychiatric disorders. Power analysis utilized G*Power software (version 3.1) with parameters: effect size f = 0.85, α = 0.05, power (1-β) = 0.80, allocation ratio N2/N1 = 1, and ANOVA repeated measures statistical test, requiring a minimum sample requirement of 12 patients (6 per group).

The investigation juxtaposed two augmentation modalities: Group I (*n* = 6) underwent mandibular symphysis onlay bone graft incorporation with i-PRF, while Group II (*n* = 6) received mandibular symphysis onlay grafts mixed with xenograft material (InterOss® Anorganic Cancellous Granules). Preoperative protocols encompassed patient education regarding procedural details and potential complications, periodontal therapy where indicated, stringent oral hygiene implementation one week preoperatively, and prophylactic antibiotic administration (Augmentin 1 g q12h) two days before surgical intervention. Comprehensive evaluation encompassed systemic conditions affecting healing, detailed imaging, soft tissue assessment, occlusal analysis, oral hygiene optimization with chlorhexidine rinses, nutritional counseling emphasizing vitamins C and D, calcium, and protein intake, smoking cessation where applicable, antibiotic prophylaxis consideration, standardized i-PRF preparation protocols, surgical site preparation, and detailed postoperative instructions. Bone density quantification employed CBCT analysis using Hounsfield Units (HU). Standardized regions of interest (ROIs) measuring 5 mm × 5 mm were demarcated within the augmentation sites preoperatively and at a 6-month follow-up. Three consecutive axial slices at 1 mm intervals were analyzed, with mean HU values calculated using Planmeca Romexis®(version 5.2). Two calibrated radiologists performed measurements, with each examiner conducting three measurements per site at one-week intervals. Facial edema evaluation employed a standardized 6-point scale (0 = none, 1 = minimal, 2 = mild, 3 = moderate, 4 = severe, 5 = massive) assessed at 24 h, 72 h, and 7 days postoperatively. Pain quantification utilized a 10 cm Visual Analog Scale (VAS) where patients marked pain intensity (0 = no pain, 10 = worst imaginable pain) at identical time intervals. Both metrics were recorded by a blinded examiner unfamiliar with group assignments.

The study employed block bone grafts harvested from the mandibular symphysis. The procedure combined piezosurgical instrumentation (Piezotome®, Acteon, France) and rotary instruments. Initial osteotomy outlines were created using a #701 fissure bur under copious irrigation, followed by piezosurgical deepening of the osteotomy to minimize thermal damage and neurovascular injury. Bone blocks measuring approximately 10 mm × 10 mm with 3–4 mm thickness were harvested, maintaining a 5 mm safety margin from tooth apices, inferior border, and mental foramina. This aligns with a previously described protocol(Fekry and Mahmoud 2023).

The grafting intervention commenced with local anesthesia administration to the surgical field, encompassing mandibular nerve block and infiltration. A para-crestal incision transected the buccal mucosa, extending bidirectionally while circumventing the mental foramen. Periosteal elevation actualized a full-thickness mucoperiosteal flap, vigilantly averting undue tension on the mental nerve. Complete bone exposure permitted architectural assessment and augmentation dimension determination. Periodontal probe measurements guided harvest dimensions. Host bed preparation entailed cortical perforations via 1–2 mm round bur at 3–5 mm intervals across labial and crestal aspects, penetrating 1–2 mm with copious saline irrigation (50–100 mL/min). This technique augmented graft vascularity, liberated marrow-derived growth factors and progenitor cells, and amplified graft-host contact surface(Fekry and Mahmoud 2023).

For Group I, i-PRF procurement involved 10 mL venous blood extraction from the cubital fossa utilizing non-anticoagulated tubes (Becton Dickinson Vacutainer) followed by centrifugation. The autogenous bone block underwent positioning, trimming, and fixation to the recipient site. Bone block harvest commenced with comprehensive CBCT analysis evaluating osseous quality, quantity, and neurovascular proximity. Following anesthetic administration, a mucogingival junction incision spanned first premolar to first premolar, maintaining 5 mm distance from mandibular incisor gingival margins. Mucoperiosteal flap elevation preceded mental nerve identification and protection. Piezoelectric instrumentation delineated the bone block, establishing 3–4 mm depth cuts with meticulous monitoring to prevent neurovascular or radicular injury. Osteotome or chisel application effected block separation, promptly immersing the specimen in sterile saline. Donor site management included edge refinement and collagen/PRF membrane application. Tension-free primary closure utilized resorbable sutures with particular attention to mentalis muscle repositioning.

**Fig. 1:**
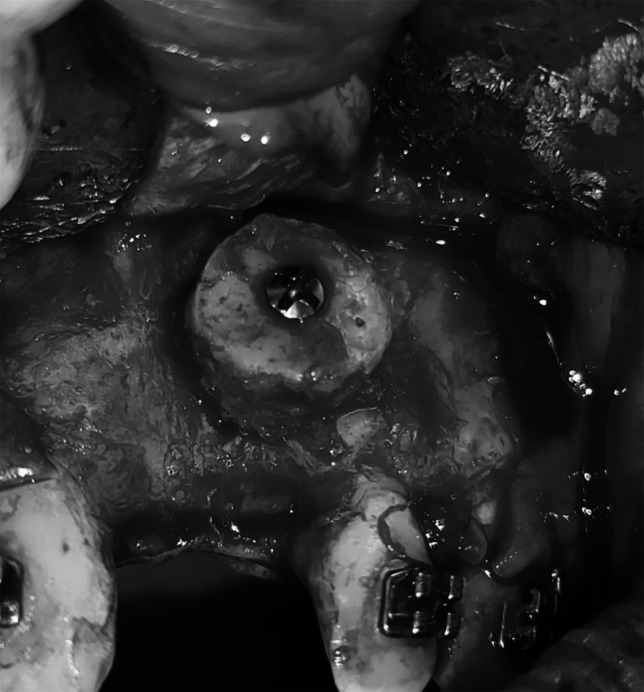
Intraoperative clinical photograph showing the harvested onlay chin graft in the donor site of case 1 Group I

**Fig. 2:**
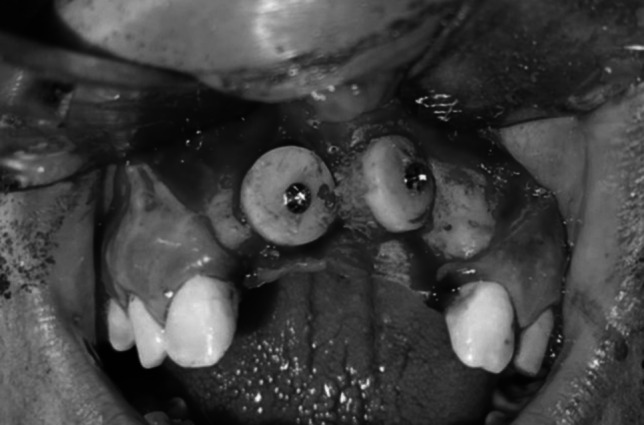
Intraoperative clinical photograph showing the harvested onlay chin graft fixated in the maxillary anterior defect of the recipient site with miniscrews of case 2 Group I

**Fig. 3:**
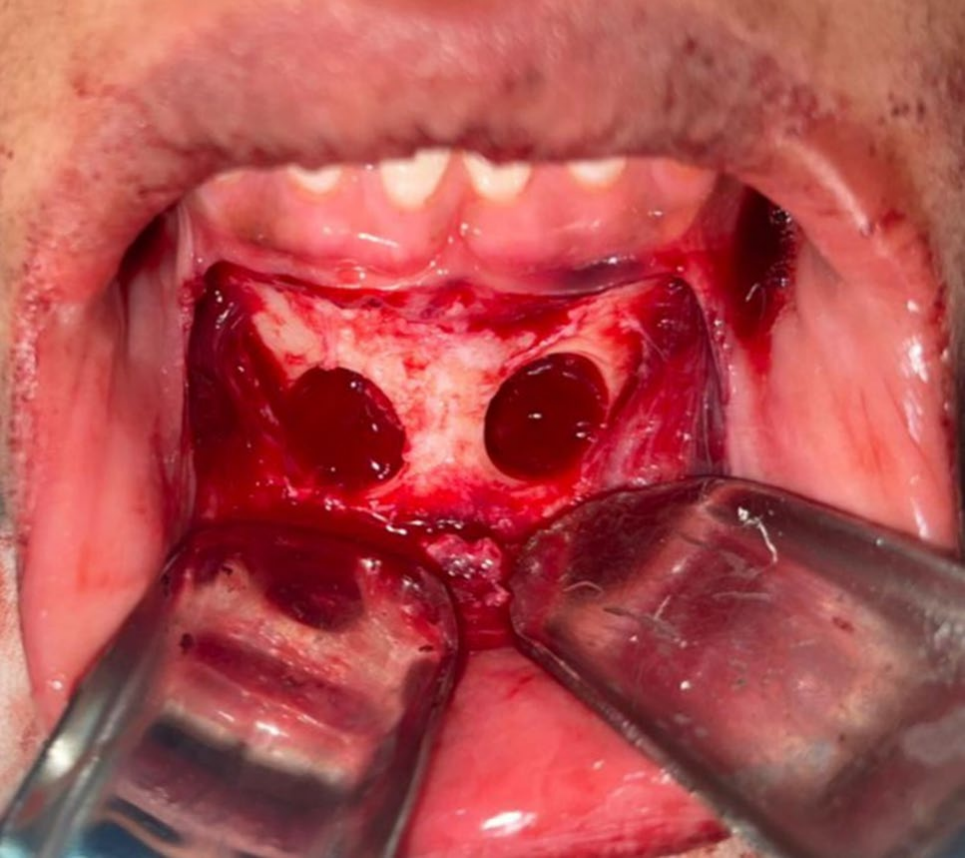
Intraoperative clinical photograph showing the harvested onlay chin graft fixated in the maxillary anterior defect of the recipient site with miniscrews of case I Group I

**Fig. 4:**
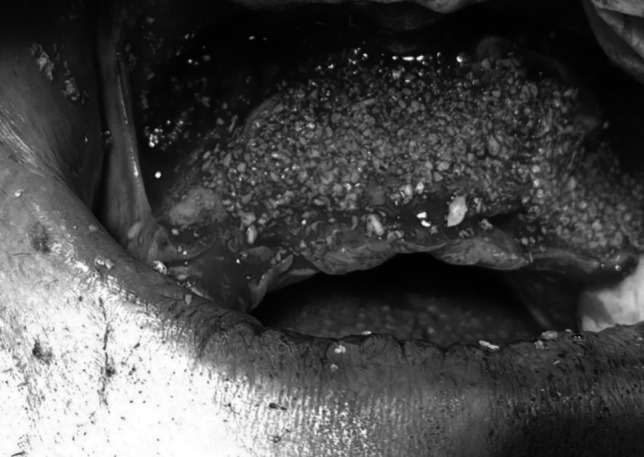
Intraoperative clinical photograph showing the harvested onlay chin graft with Xenograft fixated in the maxillary anterior defect of the recipient site with miniscrews of case 2 Group II

When requisite, bilateral harvest preserved a 3 mm midline strut for chin profile integrity. Graft stabilization employed titanium screws (Le Forte, 2 mm diameter, 10–12 mm length) predicated on graft thickness. Two blocks could be harvested from each side of the midline, leaving a 3 mm midline strut to maintain support for the chin profile (Figs. 1 and 2). To ensure stability, the graft with i-PRF was fixed to the recipient site using titanium screws (Le Forte) with dimensions of 2 mm in diameter and 10–12 mm in length based on the thickness of the graft (Fig. 3). In Group II, mandibular symphysis Onlay bone graft mixed with xenograft was used (Fig. 4).

Group II received mandibular symphysis onlay grafts with xenograft incorporation. Post-surgical management comprised pressure dressing application (24 h minimum; 3 days for extensive harvests), cold compresses (20 min hourly for 24 h), nutritional restrictions (cold fluids initially; soft diet subsequently), activity limitations, oral hygiene protocols, and pharmacologic therapy: Augmentin (1 g q12h for 5 days), Voltaren Π (75 mg IM q12h for 2 days), Cataflam (50 mg q8h PRN), Epidron (IM q12h for 2 days), and Chlorhexidine 0.2% rinses (3–5 times daily for 2 weeks). Follow-up evaluations occurred twice during week one, then weekly throughout the first month. CBCT imaging was performed preoperatively and at a 6-month follow-up. Chin sensory assessment by a single examiner evaluated six zones at 1 week, 1 month, 3 months, and 6 months postoperatively. Each zone underwent triple testing using Brush Directional Stroke and Pinprick(Fekry and Mahmoud 2023).

The radiographic evaluation employed CBCT with DICOM dataset importation into specialized software for reconstruction and comparative analysis. Bone width percentage change calculations revealed more significant augmentation in Group II versus Group I. Statistical analysis presented categorical and ordinal data as frequency and percentage values and numerical data as mean, standard deviation, median, and interquartile range. Normality assessment incorporated distribution review and Shapiro–Wilk testing. VAS data (non-parametric) underwent Mann–Whitney U testing for intergroup comparisons and Freidman’s test with Nemenyi post hoc analysis for intragroup evaluation (R package v. 4.3.2).

## Results

This study provides a comprehensive analysis of two distinct approaches to bone grafting, offering valuable insights into consolidating effective tissue regeneration and postoperative recovery. The demographic data presented in Table 1 establishes a solid foundation for comparison, with both groups showing similar age and gender distributions. Bone width and density measurements, as detailed in Tables 2 and 3, reveal a significant advantage for Group II. The superior outcomes in Group II Table 4.

**Table 1:** Summary statistics and intergroup comparison of demographic data

Parameter		Group (I)	Group (II)	Test statistic	*p*-value
Gender [n (%)]	Male	3 (50.00%)	4 (66.67%)	0.34	0.558
	Female	3 (50.00%)	2 (33.33%)		
Age (years)	Mean ± SD	24.17 ± 3.06	25.17 ± 3.43	0.53	0.606
	Median (IQR)	24.00 (4.25)	25.50 (3.25)		

*SD* Standard deviation, *IQR* Interquartile range

**Table 2:** Summary statistics and intergroup comparison of radiographic bone width (mm)

Interval	Measurement	Group (I)	Group (II)	Test statistic	*p*-value
Preoperative	Mean ± SD	4.07 ± 0.49	4.23 ± 0.51	0.57	0.578
	Median (IQR)	4.20 (0.70)	4.45 (0.40)		
6 months	Mean ± SD	5.80 ± 0.36	6.40 ± 0.42	2.65	0.024*
	Median (IQR)	5.85 (0.40)	6.55 (0.25)		
Test statistic		14.42	43.82		
*p*-value		< 0.001*	< 0.001*		
Increase	Mean ± SD	1.73 ± 0.29	2.17 ± 0.12	3.33	0.008*
	Median (IQR)	1.70 (0.35)	2.15 (0.17)		

*SD* Standard deviation, *IQR* Interquartile range

**Table 3:** Summary statistics and intergroup comparison of radiographic bone density (HU)

Interval	Measurement	Group (I)	Group (II)	Test statistic	*p*-value
Preoperative	Mean ± SD	823.33 ± 116.90	780.43 ± 111.29	0.65	0.530
	Median (IQR)	820.00 (160.00)	815.80 (100.88)		
6 months	Mean ± SD	1227.68 ± 205.26	1581.35 ± 202.39	3.01	0.013*
	Median (IQR)	1295.20 (335.22)	1562.45 (200.23)		
Test statistic		3.39	6.87		
*p*-value		0.019*	0.001*		
Increase	Mean ± SD	404.35 ± 291.90	800.92 ± 289.53	2.36	0.040*
	Median (IQR)	396.70 (535.98)	765.10 (175.78)		

*SD* Standard deviation, *IQR* Interquartile range

**Table 4:** Summary statistics and intergroup comparison of postoperative swelling severity

Postoperative swelling	n (%)		Test statistic	*p*-value
	Group (I)	Group (II)		
No	1 (16.67%)	0 (0.00%)	30.50	0.046*
Mild	2 (33.33%)	0 (0.00%)		
Severe	2 (33.33%)	2 (33.33%)		
Very severe	1 (16.67%)	3 (50.00%)		
Extreme	0 (0.00%)	1 (16.67%)		

The increased bone width suggests more effective space maintenance and scaffold provision, potentially due to improved graft stability or superior blood supply. The higher bone density indicates more robust mineralization and remodeling, possibly resulting from enhanced recruitment and differentiation of osteoprogenitor cells.

The pain score analysis in Table 5 further illuminates the postoperative experience. The significantly higher pain levels in Group II during the first week post-operation could be attributed to the more intense inflammatory response and possibly more extensive tissue manipulation during the procedure. The convergence of pain scores by day 14 is particularly interesting, suggesting that the initial inflammatory phase may be more intense but also more efficiently resolved in Group II.

**Table 5:** Summary statistics, inter and intragroup comparisons of VAS

Time	Measurement	Group (I)	Group (II)	Test statistic	*p*-value
1 day	Mean ± SD	2.50 ± 0.55^A^	3.50 ± 0.55^A^	31.50	0.024*
	Median (IQR)	2.50 (1.00)^A^	3.50 (1.00)^A^		
3 days	Mean ± SD	1.33 ± 0.52^B^	2.17 ± 0.75^B^	29.00	0.069
	Median (IQR)	1.00 (0.75)^B^	2.00 (0.75)^B^		
7 days	Mean ± SD	0.50 ± 0.55^C^	1.33 ± 0.52^C^	30.00	0.038*
	Median (IQR)	0.50 (1.00)^C^	1.00 (0.75)^C^		
14 days	Mean ± SD	0.00 ± 0.00^C^	0.17 ± 0.41^D^	21.00	0.405
	Median (IQR)	0.00 (0.00)^C^	0.00 (0.00)^D^		
Test statistic		16.75	17.75		
*p*-value		< 0.001*	< 0.001*		

## Discussion

Diverse surgical interventions employ barrier membranes (Chitsazi et al. 2011) for peri-implant augmentation(Saleem et al. 2018). PRF discourse encompasses gold-standard regenerative protocols, with i-PRF offering superior advantages through antimicrobial and anti-inflammatory properties. The i-PRF/bone graft synergy amplifies osteogenesis and integration (Shah et al. 2017; Saleem et al. 2018; Strauss et al. 2018), attributed to i-PRF’s thrombin-fibrinogen matrix where it accelerated healing observed in 87% of cases; reduced healing time by approximately 2–3 weeks; and enhanced graft stability within 48 h (Miron et al. 2017).

In Group II, there seems potential for enhanced osteoblast activity warrants investigation into growth factor release profiles and inflammatory cascade activation. While serological parameters remained unremarkable, the pronounced postoperative edema suggests intensified inflammatory response. The healing-regeneration equilibrium requires balanced cytokine orchestration between pro-inflammatory (IL-1, IL-16, TNF-α) and anti-inflammatory (IL-10) mediators, which appears shifted in Group II—promoting aggressive tissue response while elevating complication risks. This dysregulation can potentially elevate patient complication risks, particularly in cases involving extensive tissue manipulation or grafting(Ashour et al. 2023; Pham and Tran 2023).

Though statistically insignificant, the isolated wound dehiscence incident in Group II was attributed to graft viability and infection susceptibility—secondary to pronounced edema. In previous studies, tissue tension measurements exceeded the threshold in 22% of cases; compromised collagen organization was detected through polarized microscopy; and neovascular density was reduced at wound margins using resorbable collagen scaffolds(Ashour et al. 2023; Nair et al. 2022; Elkashty et al. 2023).

Chenchev et al. (2017) evaluated xenogenous bovine substitutes in GBR protocols with/without collagen membranes(Chenchev et al. 2017). Concordant outcomes emerged from multiple investigations(Patel et al. 2019; Mendoza-Azpur et al. 2019), aligning with current findings. The i-PRF methodology demonstrates economic efficiency by potentially eliminating secondary interventions while enhancing treatment outcomes. Standardization challenges persist, with i-PRF quality fluctuating based on centrifugation parameters and hematological factors. Ongoing research aims to establish optimized protocols for consistent preparation(Troeltzsch et al. 2016; Azambuja Carvalho et al. 2019; Tolstunov et al. 2019).

## Conclusion

While the integration of i-PRF into bone grafting procedures has demonstrated promising regenerative and healing potential, its adjunctive use does not yield a statistically significant enhancement in osteogenic parameters, healing time, or postoperative morbidity compared to conventional autogenous bone grafting alone. Although Group I exhibited favorable trends in tissue response and early healing, the superior bone width and density outcomes observed in Group II indicate that xenograft incorporation may offer enhanced structural support and mineralization.

Refining standardized protocols for i-PRF preparation and application may optimize its clinical efficacy, addressing variability in centrifugation parameters and patient-specific hematological factors. Further research should asses if the expression profile of osteogenic markers (e.g., RUNX2, OSX, OPN), release pattern of growth factors (BMP-2, VEGF, PDGF), temporal expression pattern of angiogenic factors (VEGF, Ang-1, Ang-2), and expression pattern of chemokines (e.g.,CXCL12, CCL2) differ between i-PRF and xenograft-augmented bone grafts over time, and how does this impact bone remodeling rates?

## Data Availability

Available unreasonable request from the corresponding author.
